# Notch Inhibition via GSI Treatment Elevates Protein Synthesis in C2C12 Myotubes

**DOI:** 10.3390/biology9060115

**Published:** 2020-06-02

**Authors:** Joshua R. Huot, Joseph S. Marino, Michael J. Turner, Susan T. Arthur

**Affiliations:** 1 Laboratory of Systems Physiology, Department of Kinesiology, University of North Carolina at Charlotte, Charlotte, NC 28223, USA; jrhuot@iu.edu (J.R.H.); jmarin10@uncc.edu (J.S.M.); miturner@uncc.edu (M.J.T.); 2 Department of Surgery, Indiana University School of Medicine, Indianapolis, IN 46202, USA

**Keywords:** protein synthesis, GSI, notch, hypertrophy

## Abstract

The role of Notch signaling is widely studied in skeletal muscle regeneration but little is known about its influences on muscle protein synthesis (MPS). The purpose of this study was to investigate whether Notch signaling is involved in the regulation of MPS. C2C12 cells were treated with a γ-secretase inhibitor (GSI), to determine the effect of reduced Notch signaling on MPS and anabolic signaling markers. GSI treatment increased myotube hypertrophy by increasing myonuclear accretion (nuclei/myotube: *p* = 0.01) and myonuclear domain (myotube area per fusing nuclei: *p* < 0.001) in differentiating C2C12 cells. GSI treatment also elevated myotube hypertrophy in differentiated C2C12s (area/myotube; *p* = 0.01). In concert, GSI treatment augmented pmTOR Ser2448 (*p* = 0.01) and protein synthesis (using SUnSET method) in myotubes (*p* < 0.001). Examining protein expression upstream of mTOR revealed reductions in PTEN (*p* = 0.04), with subsequent elevations in pAKT Thr308 (*p* < 0.001) and pAKT Ser473 (*p* = 0.05). These findings reveal that GSI treatment elevates myotube hypertrophy through both augmentation of fusion and MPS. This study sheds light on the potential multifaceted roles of Notch within skeletal muscle. Furthermore, we have demonstrated that Notch may modulate the PTEN/AKT/mTOR pathway.

## 1. Introduction

Aging and skeletal muscle-associated diseases (e.g., diabetes, cancer cachexia) can lead to significant reductions in skeletal muscle mass, which impedes quality of life and the ability for individuals to perform activities of daily living [1,2,3]. The reductions in skeletal muscle mass in these debilitating conditions are partly attributed to dysfunction in myogenesis and muscle protein synthesis (MPS). Notch signaling is a highly conserved cell-to-cell communication pathway and a key determinant of muscle satellite cell (SC) activity. Notch signaling occurs when one of its transmembrane receptors, Notch1, Notch2, Notch3, or Notch4, binds a Notch ligand (Delta-like protein (DLL)1, DLL3, DLL4, Jagged1, and Jagged2), initiating a series of metalloprotease and γ -secretase driven cleavages. The terminal γ-secretase cleavage releases Notch intracellular domain (NICD), which translocates to the nucleus as active Notch. In addition to its pivotal roles during development, Notch signaling is crucial for a successful myogenic response following injury [4,5]. Notch signaling may be dysfunctional in aged skeletal muscle, resulting in a weakened myogenic response. Dysfunctional Notch signaling has been identified as a contributor to the development of insulin resistance and cachexic muscle [6,7,8]. However, whether Notch signaling influences MPS has yet to be determined. Understanding the role of Notch on MPS will shed light on its contribution to aging and disease.

The state of Notch signaling is influential over the myogenic program. Active Notch signaling maintains SCs in a quiescent state and prevents muscle cell differentiation [9,10,11]. Inhibition of notch signaling increases differentiation and fusion of myoblasts, enhancing myotube formation [11]. Enhanced myotube formation could be considered a mode of hypertrophy. With its inhibitory role on myotube formation and size, Notch could also be considered a negative regulator, or a brake on hypertrophy. In support of this, it was demonstrated that Notch signaling drives skeletal muscle atrophy seen in cancer cachexia [7]. Despite Notch’s regulation of myogenesis and its impact on muscle mass, it is unknown whether Notch has a regulatory role on MPS. In addition, the interaction of Notch with other signaling pathways during the myogenic response and protein synthesis is not well studied.

Mechanistic target of rapamycin (mTOR) is an established regulator of MPS [12,13]. It is also postulated that mTOR regulates the latter stages of myogenesis, specifically differentiation and maturation of myotubes [14,15,16]. If Notch signaling impacts MPS, then it may be possible that mTOR signaling would be affected. Due to the antagonistic roles of Notch and mTOR on the differentiation process, it is plausible that some level of interaction between Notch and mTOR contributes to myogenic progression. T-cell leukemia cell lines and Drosophila melanogaster models of Notch induced tumorigenesis have demonstrated that Notch and Phosphatidylinositol-4,5-bisphosphate 3-kinase (PI3K) (upstream of mTOR) are linked via phosphatase and tensin homolog (PTEN), an upstream negative regulator of protein kinase B (AKT)/mTOR [17]. Specifically, it has been postulated that hairy and enhancer of split-1 (Hes1) (downstream Notch effector) regulates PTEN expression [17]. However, it is unknown whether Notch signaling impacts the PTEN/AKT/mTOR pathway in skeletal muscle thereby influencing MPS. Here we demonstrate that Notch inhibition in C2C12 mouse muscle cells, elevates MPS along with the AKT/mTOR pathway.

## 2. Materials and Methods

### 2.1. Cell Culture

The C3H murine cell line C2C12 (ATCC p3-p8) were used for all experiments. For myoblast experiments, cells were seeded in growth media (GM: Dulbecco’s Modified Eagles Medium [DMEM], 10% fetal bovine serum (FBS), 10% horse serum (HS), and 1% penicillin/streptomycin (P/S)) in 12-well plates at a density of 10,000 cells/well. After 24 h following seeding (~30% confluence), cells were washed with phosphate buffered saline (PBS) 1 × and treated with either 4 µm γ-secretase inhibitor (GSI: L-685,458; Millipore Sigma- dimethyl sulfoxide [DMSO]), or control (DMSO equal volume to 4 µm-GSI) for 48 h (every 12 h). Following GSI treatment, myoblasts were assessed for protein expression and protein synthesis as detailed below. For GSI myotube experiments, cells were seeded in GM in 6-well plates at a density of 75,000 cells/well, grown to ~100% confluence, washed 2 × with PBS, and differentiated for 96 h in differentiation media (DM: DMEM: 2% HS, 1% P/S) with or without 4 µm —GSI every 12 h. Myotubes were analyzed for indices of fusion, protein expression, and protein synthesis as detailed below. For late-stage myotube experiments, cells were seeded, grown to ~100% confluence, washed 2 × with PBS, and differentiated for 120 h in DM. At 120 h, wells were treated with or without 4 µm-GSI every 12 h for 24 h. Next, 144—h myotubes were analyzed for indices of fusion and protein synthesis as detailed below.

### 2.2. Myosin Heavy Chain Staining

Following 96 and 144 h of differentiation, myotubes were labeled with myosin heavy chain (MHC) to determine properties of fusion and area. Briefly, cells were fixed with 70% Acetone/30% Methanol for 10 min at room temperature. Following fixation, cells were washed 2 × with PBS, blocked for 1 h with 10% normal goat serum (NGS) in PBS, and incubated at 4 °C overnight in MHC. Following overnight incubation (16 h), cells were washed 3 × with PBS, and counter stained with a secondary antibody (1:500) specific to the MHC primary and 4′,6-Diamidino-2-Phenylindole, Dihydrochloride (DAPI 1:1000) in PBS for 1 h. Wells were mounted with Vectashield and a coverslip.

### 2.3. Myotube Fusion and Myotube Area

Following MHC labeling, myotubes were imaged with an Olympus iX inverted microscope at 20× Images from wells were all taken in the same fashion: (1) the top left of the well was imaged, (2) the stage was moved over three fields of view to the right and imaged, (3) the stage was moved down three fields of view and imaged, (4) the stage was moved over three fields of view to the left and imaged, (5) the stage was moved down three fields of view and imaged, and (6) the stage was moved three fields of view to the right and imaged. Six fields of view per well were captured. Myotube fusion was determined by counting the total nuclei, total myotubes, and nuclei fusing into myotubes, by 2 blinded individuals using ImageJ software (cell counter plugin). A myotube was defined as an MHC labeled cell containing two or more nuclei. All myosegments (MHC labeled without two or more nuclei) were ignored as myotubes in the fusion index and area quantification. Fusion index was calculated by nuclei within myotubes/total nuclei. Myotube area were determined from the same images used to calculate fusion using Adobe Photoshop as previously described [18]. Briefly, three random control and three random GSI images were used to set a color range (accepted tones of red (for MHC) and blue (for DAPI)). The established color range was then applied to all experiment images, a measurement scale was set (pixels to microns), and measurements were obtained for total myotube area per field, area per myotube per field (total myotube area/number of myotubes present in a field of view), and myotube area per fused nuclei (total myotube area/number of nuclei fused into myotubes in a field of view).

### 2.4. Protein Synthesis

For protein synthesis measurements, the established and validated SUnSET method using puromycin was used as previously described [19,20]. Briefly, 30 min prior to collection, C2C12 cells were treated with 1µm puromycin (P-1033, A.G. Scientific). Puromycin incorporation was determined via Western blot analysis as described below.

### 2.5. Western Blot

Collection and preparation of C2C12s was done as previously described for Western blot analysis [21]. Briefly, C2C12 myoblasts and myotubes were washed 2× with ice cold PBS followed by addition of ice cold Radioimmunoprecipitation assay (RIPA) buffer (sc-24948; Santa Cruz, supplemented with 1% Triton-x, 2% SDS, protease cocktail inhibitor) for 5 min. Wells were scraped, mechanically lysed (using a 25 gauge-needle-syringe), and centrifuged for 20 min at 20,000 G (4 °C). Protein concentration of the supernatant was determined by Pierce BCA kit (23225; ThermoFisher). Then, 20 µg of sample was loaded onto a 4%–12% BisTris gel (3450125; Bio-rad) and run (XT MES running buffer; 1610789; Bio-rad) at 125 V for 2 h. Following electrophoresis, proteins were transferred (Towbin Buffer; 10% methanol) onto a 22 µm Polyvinylidene difluoride (PVDF) membrane for 1 h at 100 V. Membranes were washed 1× in Tris-buffered saline (TBS) and blocked for 1 h in Odyssey blocking buffer 1:1 with TBS. Following blocking, membranes were incubated overnight (16 h at 4 °C) in primary antibodies. Antibodies used included: Hes1 (#11988; 1:500), Cleaved Notch (#4147; 1:500), PTEN (#9188; 1:1000), pAKT Thr308 (#13038; 1:500), pAKT Ser473 (#4060; 1:500), AKT (#2920; 1:1000), pTSC2 Ser939 (#3615; 1:500), pTSC2 Thr1462 (#3617; 1:500), TSC2 (#4308, 1:1000), pmTOR Ser2448 (#5536; 1:500), pmTOR Ser2481 (#2974; 1:500), mTOR (#4517, 1:500), p4EBP1 Thr37/46 (#2855; 1:500), 4EBP1 (#9644; 1:1000), pp70S6K Thr389 (#92434; 1:500), p70S6K (#2708; 1:1000), peEF2 Thr56 (#2331; 1:500), and eEF2 (#2332; 1:1000) from cell signaling; Myosin Heavy Chain (#MF-20; 1:50) from Developmental Studies Hybridoma Bank; Myogenin (#82843; 1:500) from ABcam; Puromycin (#MABE343; 1:5000) from EMD Millipore; and β-Actin (#A2228; 1:10,000) from Sigma Aldrich). The next day, membranes were washed 3 × 5 min in TBST (TBS: 0.1% Tween 20) and then incubated in secondary antibodies (1:10,000 in TBST) for 1 h. Following 3 × 5 min washes in TBST and 1 × 5 min wash in TBS, membranes were imaged and bands were quantified using the Odyssey^®®®^ Licor CLx System.

### 2.6. Statistical Analysis

T-tests (two-tailed) were used to determine differences between control and GSI groups for myoblast and myotube protein expression levels as well as properties of myotube formation (Fusion/Area). Significance was set at an alpha level of 0.05. All statistics were performed using GraphPad Prism 7.03 and all data are presented as means ± SD.

## 3. Results

### 3.1. GSI Increases C2C12 Myotube Formation and Hypertrophy

**Figure 1:**
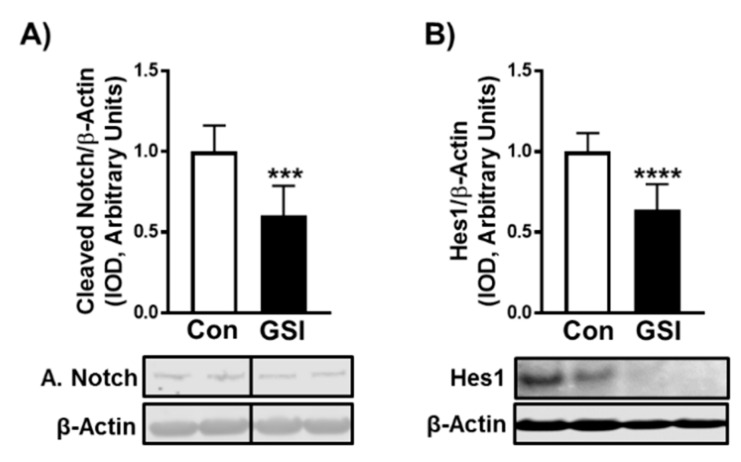
GSI reduces Notch signaling in differentiating C2C12 myotubes. (**A**) NICD/β-Actin; (**B**) Hes1/β-Actin expression (Integrated optical density, IOD) in 96-h myotubes treated with or without 4 µm γ-secretase inhibitor (GSI) every 12 h. Thirty minutes prior to collection all cells were treated with 1µm puromycin. For representative image: lanes 1 and 2 are Con; lanes 3 and 4 are GSI. Data were analyzed using a Student’s T-test. *** *p* < 0.001 vs. Control (Con). **** *p* < 0.0001 vs. Con (*n* = 3 experiments). Data are mean ± SD.

**Figure 2:**
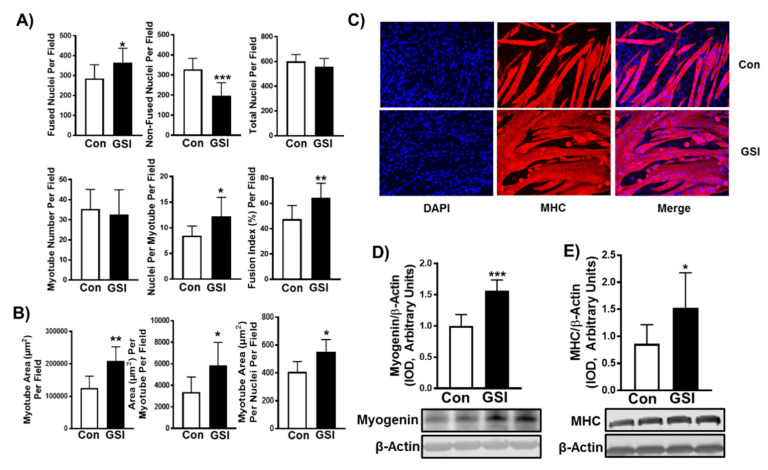
GSI increases myotube formation in differentiating C2C12 myotubes. (**A**) Indices of myotube fusion. Graph order, top left to right: Fused nuclei per field, Non-fused nuclei per field, and Total nuclei per field. Graph order, bottom left to right: Myotube number per field, Nuclei per myotube per field, Fusion index per field. (**B**) Indices of myotube hypertrophy. Graph order: Myotube area (µm) per field, Area (µm) per myotube per field, Myotube area (µm) per nuclei per field. (**C**) Representative image of 96-h myotubes co-stained with myosin heavy chain (MHC:red) and DAPI:blue. Images were taken at 20× magnification and the scale bar = 50 µm. (**D**) Myogenin/β-Actin; (**E**) MHC/β-Actin expression (Integrated optical density, IOD) in 96-h myotubes treated with or without 4 µm γ-secretase inhibitor (GSI) every 12 h. For Western blot representative images: lanes 1 and 2 are Con; lanes 3 and 4 are GSI. Thirty minutes prior to collection all cells were treated with 1µm puromycin. Data were analyzed using a Student’s T-test. * *p* ≤ 0.05 vs. Control (Con); ** *p* < 0.01 vs. Con; *** *p* < 0.001 vs. Con (*n* = 3 experiments). Data are mean ± SD.

Notch signaling can be reduced by utilizing γ-secretase inhibitors (GSIs), which prevent NICD (active Notch) cleavage. To determine the impact that GSI treatment had on myotube formation and hypertrophy, C2C12 cells were treated for 96 h with or without 4 µm of GSI. The 4 µm GSI treatment reduced NICD by ~40% (*p* < 0.001) and Hes1 expression by ~38% (*p* < 0.0001) in C2C12 myotubes (Figure 1A,B) compared to control (Con: DMSO). As anticipated, GSI treatment led to significant increases in myotube formation. All fusion markers were enhanced with GSI treatment including: fused nuclei (*p* = 0.04), non-fused nuclei (*p* < 0.001), nuclei per myotube (*p* = 0.02), and fusion index (nuclei fusing/total nuclei) (*p* = 0.008) (Figure 2A,C). There was no change in total nuclei (*p* = 0.19) or myotube number with GSI treatment (*p* = 0.62) (Figure 2A,C). In addition to increasing myotube fusion, GSI treatment increased total myotube area (*p* = 0.004), area per myotube (*p* = 0.04), and myonuclear domain (myotube area per fusing nuclei; *p* = 0.01) (Figure 2B,C). In support of these findings, myogenin (*p* < 0.001) and myosin heavy chain (*p* = 0.02) protein levels were elevated following GSI treatment (Figure 2D,E). To verify the hypertrophic impact of GSI-treated differentiating C2C12 cells, we measured indices of fusion and hypertrophy in 144-h myotubes treated with GSI for 24 h. GSI treatment did not significantly change (*p* > 0.05) fused nuclei, non-fused nuclei, total nuclei, or fusion index (Figure S1A,C). Though myotube number was not significantly different, nuclei per myotube was significantly increased (*p* = 0.02) with GSI treatment (Figure S1A,C). Neither total myotube area (*p* = 0.19) or myotube area per nuclei (*p* = 0.48) were increased with GSI treatment (Figure S1B,C). However, area per myotube was significantly increased (*p* = 0.01) with GSI treatment in 144-h myotubes (Figure S1B,C).

### 3.2. GSI Increases Protein Synthesis and mTOR Signaling in C2C12 Myotubes

**Figure 3:**
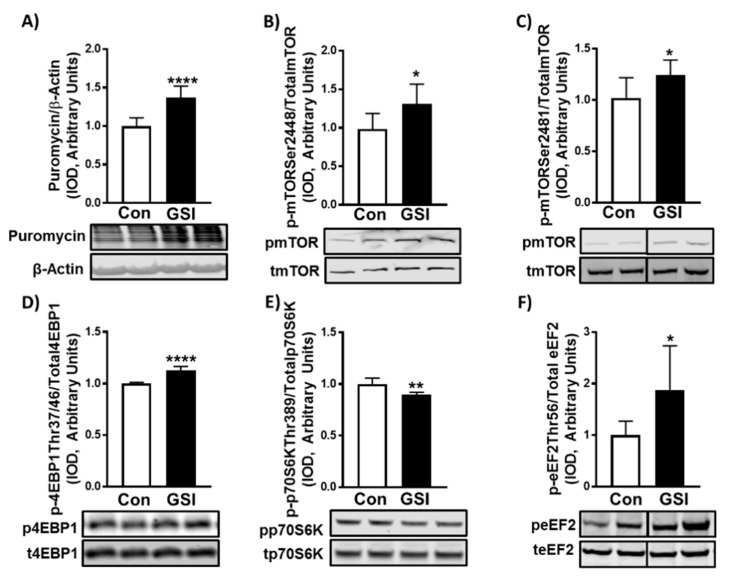
GSI increases protein synthesis and mTOR signaling in C2C12 myotubes. (**A**) Puromycin/β-Actin; (**B**) Phospho (p)-mTOR Ser2448/Total mTOR; (**C**) p-mTOR Ser2481/Total mTOR; (**D**) p-4EBP1 Thr37/46/Total 4EBP1; (**E**) p-p70S6K Thr389/Total p70S6K; (**F**) p-eEF2 Thr56/Total eEF2 expression (Integrated optical density, IOD) in 96-h myotubes treated with or without 4 µm γ-secretase inhibitor (GSI) every 12 h. Thirty minutes prior to collection all cells were treated with 1 µm puromycin. For representative images: lanes 1 and 2 are Con; lanes 3 and 4 are GSI. Data were analyzed using a Student’s T-test. * *p* ≤ 0.05 vs. Control (Con); ** *p* < 0.01 vs. Con; **** *p* < 0.0001 vs. Con (*n* = 3 experiments). Data are mean ± SD.

Coinciding with its hypertrophic effect on myotubes, GSI treatment elevated protein synthesis in 96 and 144 h-myotubes (*p* < 0.0001; *p* < 0.01) (Figure 3A and Figure S2). In line with elevations in protein synthesis, GSI treatment increased phosphorylation (Ser2448; *p* = 0.01 and Ser2481; *p* = 0.02) of mTOR (Figure 3B,C) and its downstream effector: eukaryotic initiation factor 4E binding protein (4EBP1) (Thr37/46; *p* = 0.0001) (Figure 3D) in 96-h myotubes. Intriguingly, phosphorylation of 70 kDa ribosomal protein S6 kinase (p70S6K) (Thr389) and eukaryotic elongation factor 2 (eEF2) (Thr56) were decreased (*p* = 0.002) and increased (*p* = 0.037), respectively (Figure 3E,F). Additionally, GSI treatment also increased protein synthesis in C2C12 myoblasts (*p* < 0.0001; Figure S3A). These same experiments revealed increases in phosphorylation of mTOR at Ser2448 (*p* = 0.004) and Ser2481 (*p* = 0.02) (Figure S3B,C), and its downstream effector 4EBP1 (Thr37/46; *p* = 0.03) (Figure S3D). Interestingly, there was no significant difference in either p70S6K (Thr389; *p* = 0.81) (Figure S3E) or eEF2 (Thr56; *p* = 0.29) (Figure S3F) in myoblasts.

### 3.3. GSI Increases Signaling Upstream of mTOR

**Figure 4:**
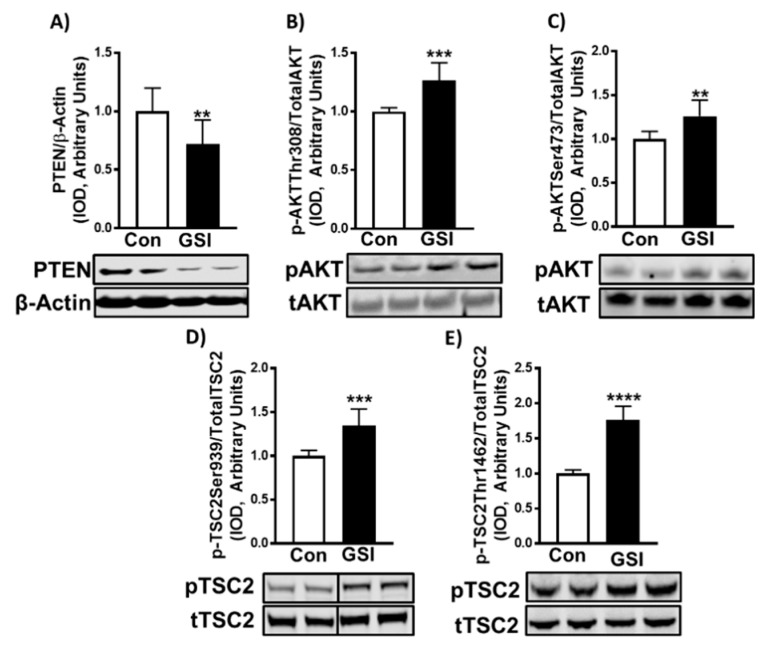
GSI increases signaling upstream of mTOR in C2C12 myotubes. (**A**) PTEN/β-Actin; (**B**) p-AKT Thr308/Total AKT; (**C**) p-AKT Ser473/Total AKT; (**D**) p-TSC2 Ser939/Total TSC2; (**E**) p-TSC2 Thr1462/Total TSC2 expression (Integrated optical density, IOD) in 96-h myoblasts treated with or without 4 µm γ-secretase inhibitor (GSI) every 12 h. Thirty minutes prior to collection all cells were treated with 1 µm puromycin. For representative image: lanes 1 and 2 are Con; lanes 3 and 4 are GSI. Data were analyzed using a Student’s T-test. ** *p* < 0.01 vs. Control (Con); *** *p* < 0.001 vs. Con; **** *p* < 0.0001 (*n* = 3 experiments). Data are mean ± SD.

We then tested if inhibition of Notch alters the expression of proteins upstream of mTOR, including PTEN, AKT, and tuberous sclerosis complex 2 (TSC2). GSI treatment reduced PTEN (*p* = 0.009) (Figure 4A) expression and increased phosphorylation of AKT (Thr308; *p* < 0.001 and Ser473; *p* = 0.001) and TSC2 (Ser939; *p* < 0.001 and Thr1462; *p* < 0.0001) (Figure 4B–E) in 96-h myotubes. Mimicking its effect in myotubes, 4 µm GSI-treatment reduced PTEN expression in myoblasts (*p* < 0.001; Figure S4A). Additionally, GSI treatment increased phosphorylation of AKT (Thr308; *p* < 0.001 and Ser473; *p* < 0.001) and TSC2 (Ser939; *p* = 0.03 and Thr1462; *p* = 0.01) (Figure S4B–E).

## 4. Discussion

Though Notch signaling has been widely studied within skeletal muscle, a majority of this research has focused on Notch’s role in the regulation of SC activity. Previous findings demonstrated that inhibition of Notch signaling induced muscle cell differentiation, enhanced myotube formation, and led to hypertrophy [7,22]. Despite extensive research on Notch signaling within skeletal muscle, to our knowledge, we have been the first to investigate its impact on in vitro muscle growth via MPS. Here we report that Notch inhibition (via GSI treatment) elevates MPS in C2C12 muscle cells.

Signaling of the Notch family transmembrane receptors, Notch1 through Notch4, occurs when ligands (DLL 1, DLL 3, DLL4, Jagged1, and Jagged2) bind and initiate a series of cleavages driven by metalloproteases and γ-secretases, ending with release and subsequent translocation of NICD to the nucleus. Translocated NICD induces expression of several target genes including Hes1, hairy/enhancer-of-split related with YRPW motif protein 1 (Hey1), and avian myelocytomatosis viral oncogene homolog (MYC) [2,23]. Notch’s influence over SC activation and myoblast proliferation via regulation of myogenic regulatory factors (e.g., Pax7, MyoD) has been widely studied [9,24]. However, specific roles for Notch and its downstream effectors within other skeletal muscle processes such as MPS are largely unknown.

A commonly used method of inhibiting Notch signaling is treating cells with a GSI. Here we demonstrated that GSI treatment reduced NICD and Hes1 in C2C12 myotubes compared to control (Figure 1). Our data support prior findings that Notch signaling has an inhibitory effect on myotube formation and expression of the late-stage myogenic regulatory factors, myogenin and myosin heavy chain (Figure 2A–E) [5,24,25]. However, the exact mechanism by which Notch inhibition elevates myotube formation is unknown as several Notch effectors (Hes1/MyoD, Hey, MyoR (Musculin)) have shown to suppress the myogenic response [26,27]. Despite evidence of mTOR’s roles in SC activation, differentiation, and fusion and Notch’s known ability to halt differentiation and fusion, interplay between these two signaling pathways within skeletal muscle has not been elucidated [14,28,29]. GSI treatment increased myotube fusion (e.g., fusion index, nuclei/myotube) and mTOR signaling (Figure 2A–C and Figure 3B–C). Therefore, mTOR may be an additional mechanism by which Notch mediates myoblast differentiation and fusion. However, future studies must investigate if the enhanced fusion effects of GSI are dependent on mTOR.

Maintaining or increasing skeletal muscle mass (hypertrophy) is important for sustaining quality of life in aging and diseased populations. Post-natal hypertrophy is achieved by increasing cellular fusion (myonuclear accretion) or by increasing protein synthesis (expansion of myonuclear domain) [30,31,32]. Given what is known about Notch’s regulation over SCs and prevention of differentiation, it is no surprise that Notch inhibition mediated myotube hypertrophy by increasing cellular fusion (Figure 2). In addition to an increase in myonuclear accretion we also show expanded myonuclear domain, by which myotube area per fused nuclei increased with GSI treatment (Figure 2B). Our findings are in concert with previous literature showing that mutations in Notch associated proteins (Protein O–Fucosyltransferase 1 (Pofut1)) induced in vitro and in vivo muscle hypertrophy [33,34]. In contrast, when Notch-associated proteins are deleted during development it induces premature differentiation resulting in hypotrophy [35,36]. Given the specific roles of Notch signaling, it is possible that Notch levels are essential to sustain proper muscle development, but that down regulation of Notch in developed post-natal muscle allows for hypertrophy. Our findings revealed that Notch may serve as a molecular brake on myotube hypertrophy through regulation of both myonuclear accretion (fusion) and expansion of myonuclear domain (protein synthesis). It is plausible that the increased cellular fusion was the primary driver of myotube hypertrophy. However, we believe that Notch warrants further investigation in its role of expansion of myonuclear domain. We wanted to further examine if GSI had similar effects on already formed myotubes (treatment starting at hour 120 of differentiation). The effect of GSI on 144-h myotubes was not as robust compared to when C2C12 cells were treated from the onset of differentiation. However, area per myotube and nuclei per myotube were elevated (Figure S1A–C). Both changes coincide with the slight non-significant reduction in myotube number. This data suggests that the myotubes were increasing in size ultimately by fusing together, yielding the increase in area per myotube. These findings suggest that GSI treatment may be able to increase hypertrophy of mature skeletal muscle.

Increasing protein synthesis is pivotal to maintaining lean body mass in aged and diseased populations. The known driver of protein synthesis, mTOR, is under vast regulation by upstream proteins including AKT and PTEN [12]. However, whether Notch signaling modulates MPS has yet to be elucidated. We demonstrated that Notch via use of a GSI modulates protein synthesis in C2C12 skeletal muscle cells (Figure 3A and Figure S3A). In both proliferating myoblasts and differentiating myotubes GSI treatment significantly elevated protein synthesis. This elevation of protein synthesis is in concert with our findings of increased myonuclear domain in C2C12 myotubes (myotube area/fusing nuclei). Also of interest, the treatment of myotubes at 120 h post-differentiation increased MPS, suggesting that Notch inhibition may also be able to augment MPS in post-natal skeletal muscle (Figure S2). Interestingly, with all the evidence pointing to modulation of differentiation and fusion leading to hypertrophy, this was, to our knowledge, the first study investigating the possibility of Notch’s role mediating hypertrophy via protein synthesis.

In addition to showing elevations in mTOR signaling and protein synthesis, we demonstrated that GSI treatment may act on mTOR via modulation of the PTEN/AKT cascade (Figure 4 and Figure S4). PTEN is a negative upstream regulator of AKT and mTOR. It does so by dephosphorylating Phosphatidylinositol (3,4,5)-trisphosphate (PIP3) to Phosphatidylinositol 4,5-bisphosphate (PIP2) [12]. Here we revealed that in both myoblasts and myotubes, PTEN expression is significantly reduced following GSI treatment. Following suit, we demonstrated increased phosphorylated AKT (Thr308 and Ser473) and phosphorylated TSC2 (Ser939 and Thr1462) with GSI treatment (Figure 4 and Figure S4). Research outside of the skeletal muscle field supports the notion that Notch may regulate the PTEN/AKT/mTOR cascade. Within T-cell acute lymphoblastic leukemia (T-ALL), Notch signaling has demonstrated modulation over phosphatidylinositol 3-kinase (PI3K), AKT, and mTOR [17,37,38,39]. Both Hes1 and C-myc have shown to target PTEN expression [17], while C-myc has also been identified to regulate gene expression of TSC2 [40]. Along with the results presented here, prior literature supports Notch as a plausible modulator of the PI3K/AKT/mTOR cascade. In addition, our data revealed increased P-AKT Ser473 in myoblasts and myotubes exposed to GSI, which indicates activity in the mTORC2 complex. In support of this, we showed elevations of P-mTOR Ser2481, which has been implicated as a surrogate for mTORC2 activity [41]. Interestingly, non-canonical roles for NICD have been identified in which NICD integrates with mTORC2 to regulate AKT-mediated (P-AKTSer473) antiapoptotic effects [42].

To our knowledge we are the first to address the research paradigm that Notch may regulate MPS. Though we demonstrated that use a GSI is sufficient to elevate MPS as well as the AKT/mTOR axis, this study is not without limitations. GSIs are a common approach to inhibit Notch signaling, however they are known to effect other signaling pathways [22]. Moreover, GSIs do not selectively target the Notch receptors, therefore future studies should investigate whether specific Notch receptors are involved in MPS, through use of gene targeting approaches. Lastly, though we have shown GSI treatment increases MPS and AKT/mTOR, the present experiments did not determine whether elevations in MPS via GSI were dependent on AKT/mTOR. This is of importance as we did not observe normative signaling downstream of mTOR. Under activated conditions, mTOR will usually result in increased phosphorylation of 4EBP1 and p70S6K, while eEF2 will be dephosphorylated resulting in increased MPS [12]. Despite increased phosphorylation of AKT and mTOR, only 4EBP1 was phosphorylated in a pro-MPS manner whereas p70S6K and eEF2 were either not effected (myoblasts) or altered in an anti-MPS manner (myotubes). Given that we saw modulation of the PTEN/AKT axis, with increased phosphorylation of AKT at both threonine 308 and serine 473, it is possible that the increased MPS observed was acting primarily via AKT. Though AKT has been identified as a modulator of mTOR dependent-MPS, it is also able to elevate MPS independent of mTOR through its phosphorylation (Ser9) and inactivation of glycogen synthase kinase 3 beta (GSK3β) [43,44]. Under active conditions GSK3β inhibits the translation initiation factor eukaryotic initiation factor 2B (eIF2B), in particular the catalytic epsilon subunit, eIF2Bε (via phosphorylation) [44,45]. When phosphorylated by AKT on Ser9, GSK3β activity is reduced and its inhibitory phosphorylation on eIF2Bε subsides allowing for an increase in eIF2Bε functionality and subsequent translation initiation [46]. As this signaling cascade was not isolated in the present study, we can only speculate that the elevations in MPS with GSI treatment are attributed primarily to AKT. Thus, future investigations should further interrogate the specific mechanisms by which GSI treatment elevates MPS and whether the effects are dependent upon AKT and/or mTOR.

## 5. Conclusions

This study further elucidated the molecular braking effect that Notch signaling has on skeletal muscle. We demonstrated that GSI treatment increases protein synthesis in C2C12 muscle cells. The increased protein synthesis, increased myonuclear domain, and increased myonuclear accretion indicate that Notch may modulate skeletal muscle hypertrophy via multiple avenues. Future research should focus on further elucidating the regulation of protein synthesis by Notch signaling and whether Notch signaling can modulate MPS in vivo.

## Supplementary Materials

The following are available online at https://www.mdpi.com/2079-7737/9/6/115/s1, Figure S1: GSI augments myotube hypertrophy in day 6 myotubes, Figure S2: GSI augments protein synthesis in day 6 myotubes, Figure S3: GSI elevates protein synthesis and mTOR signaling in C2C12 myoblasts, Figure S4: GSI modulates signaling upstream of mTOR in C2C12 myoblasts.
