# Supplementary material for: Notch Inhibition via GSI Treatment Elevates Protein Synthesis in C2C12 Myotubes
Source: Biology (Basel). 2020 Jun 2;9(6):115. doi: 10.3390/biology9060115 (PMC7345243; doi:10.3390/biology9060115)
Supplement: Supplementary file 1 [file biology-09-00115-s001.zip › biology-796884-supplementary-final/biology-796884-supplementary-final.docx]

Supplementary

Notch Inhibition via GSI Treatment Elevates Protein Synthesis in C2C12 Myotubes

Joshua R. Huot, Joseph S. Marino, Michael J. Turner and Susan T. Arthur


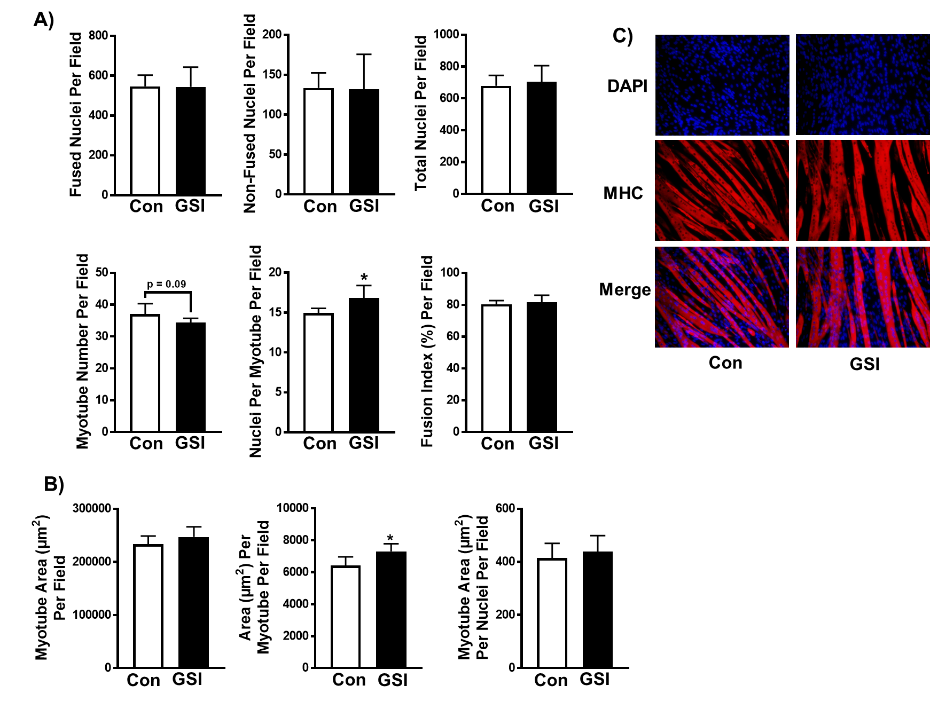


**Figure S1. GSI augments myotube hypertrophy in day 6 myotubes.** (**A**) Indices of myotube fusion. Graph order: Graph order: (top) Fused nuclei per field, Non-fused nuclei per field, Total nuclei per field, (bottom) Myotube number per field, Nuclei per myotube per field, Fusion index per field. (**B**) Indices of myotube hypertrophy. Graph order: Myotube area (µm) per field, Area (µm) per myotube per field, Myotube area (µm) per nuclei per field. (**C**) Representative image of 144-h myotubes co-stained with myosin heavy chain (MHC) and DAPI. Images were taken at 20x magnification and the scale bar = 50 µm. 120-h post differentiation C2C12 cells were treated every 12 h with either control (Con) or 4 µm γ-secretase inhibitor (GSI). Data were analyzed using a Student’s T-test. * *p* < 0.05 vs. Con. (*n* = 2 experiments). Data are mean ± SD.


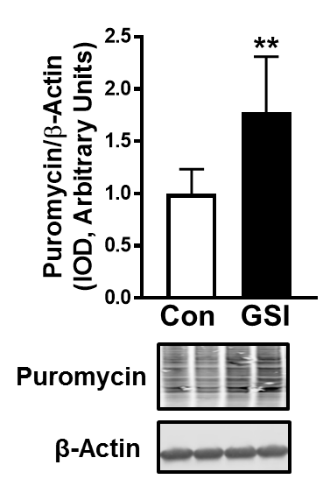


**Figure S2. GSI augments protein synthesis in day 6 myotubes.** Puromycin/β-Actin expression (Integrated optical density, IOD) in 144-h myotubes. 120-h post differentiation C2C12 cells were treated every 12 h with either control (Con) or 4 µm γ-secretase inhibitor (GSI). Thirty minutes prior to collection all cells were treated with 1 µm puromycin. For representative image: lanes 1 and 2 are Con; lanes 3 and 4 are GSI. Data were analyzed using a Student’s T-test. ** *p* ≤ 0.01 vs. Control (Con) (*n* = 3 experiments). Data are mean ± SD.


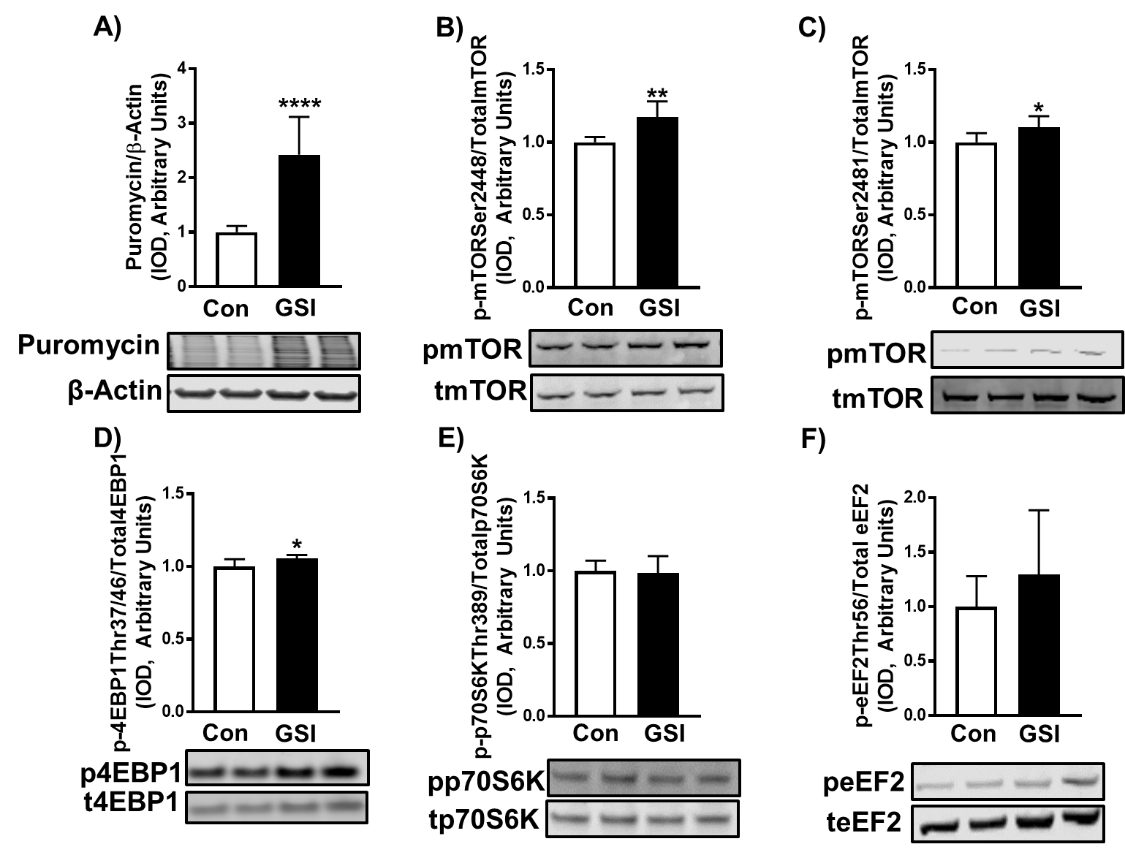


**Figure S3. GSI elevates protein synthesis and mTOR signaling in C2C12 myoblasts.** (**A**) Puromycin/β-Actin; (**B**) p-mTOR Ser2448/Total mTOR; (**C**) p-mTOR Ser2481/Total mTOR; (**D**) p-4EBP1 Thr37/46/Total 4EBP1; (**E**) pp70S6K Thr389/Total p70S6K; (**F**) p-eEF2 Thr56/Total eEF2 expression (Integrated optical density, IOD) in 48-h myoblasts treated with or without 4 µm γ-secretase inhibitor (GSI) every 12 h. Thirty minutes prior to collection all cells were treated with 1 µm puromycin. For representative image: lanes 1 and 2 are Con; lanes 3 and 4 are GSI. Data were analyzed using a Student’s T-test. * *p* ≤ 0.05 vs. Control (Con); ** *p* < 0.01 vs. Con; **** *p* < 0.0001 vs. Con (*n* = 3 experiments). Data are mean ± SD.


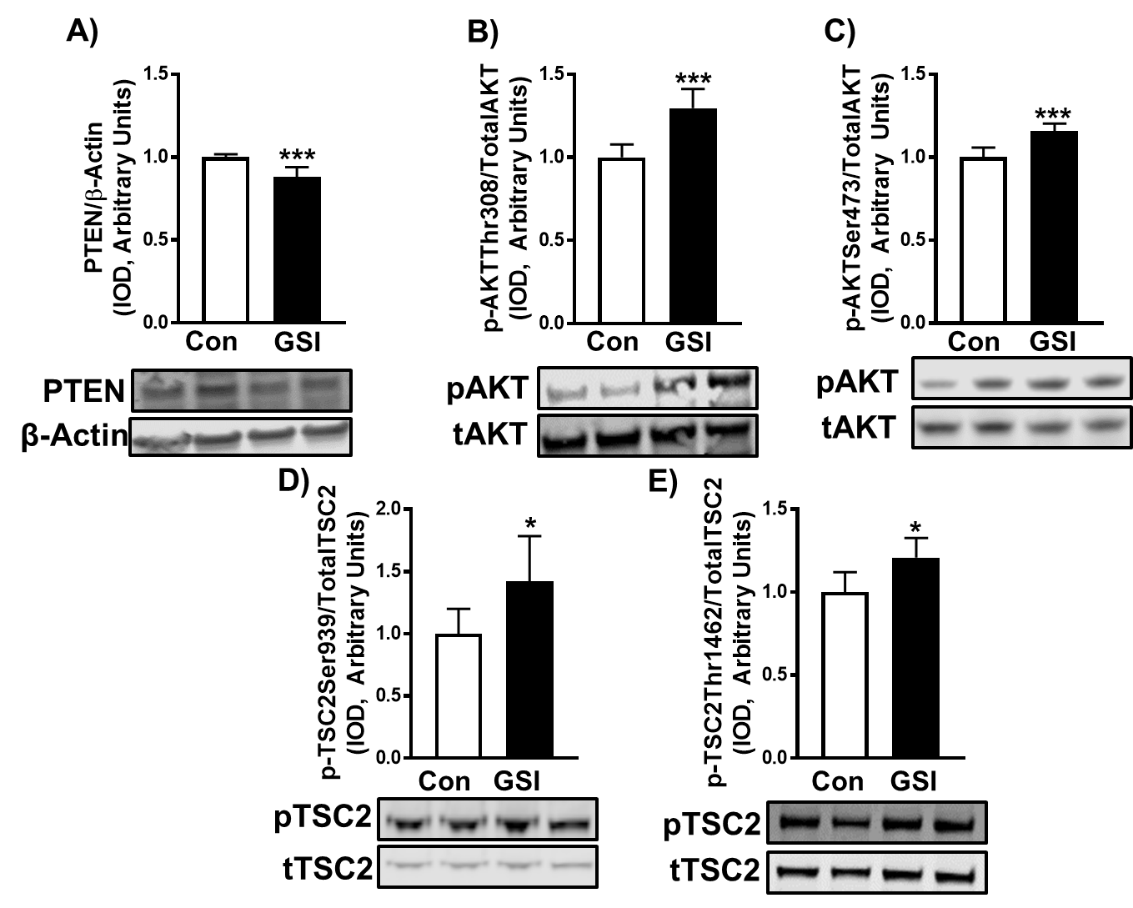


**Figure S4. GSI modulates signaling upstream of mTOR in C2C12 myoblasts.** (**A**) PTEN)/β-Actin; (**B**) p-AKT Thr308/Total AKT; (**C**) p-AKT Ser473/Total AKT; (**D**) p-TSC2 Ser939/Total TSC2; (**E**) p-TSC2 Thr1462/Total TSC2 expression (Integrated optical density, IOD) in 48-h myoblasts treated with or without 4 µm γ-secretase inhibitor (GSI) every 12 h. Thirty minutes prior to collection all cells were treated with 1 µm puromycin. For representative image: lanes 1 and 2 are Con; lanes 3 and 4 are GSI. Data were analyzed using a Student’s T-test. * *p* ≤ 0.05 vs. Control (Con); *** *p* < 0.001 vs. Con (*n* = 3 experiments). Data are mean ± SD.

| 1. 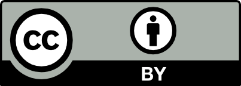 | 1. © 2020 by the authors. Submitted for possible open access publication under the terms and conditions of the Creative Commons Attribution (CC BY) license (http://creativecommons.org/licenses/by/4.0/). |
| --- | --- |
