# Supplementary material for: Notch Inhibition via GSI Treatment Elevates Protein Synthesis in C2C12 Myotubes
Source: Biology (Basel). 2020 Jun 2;9(6):115. doi: 10.3390/biology9060115 (PMC7345243; doi:10.3390/biology9060115)
Supplement: Supplementary file 1 [file biology-09-00115-s001.zip › biology-796884-supplementary-final/biology-796884-supplementary-final.pdf]

Supplementary

Notch Inhibition via GSI Treatment Elevates Protein Synthesis in C2C12 Myotubes

Joshua R. Huot, Joseph S. Marino, Michael J. Turner and Susan T. Arthur

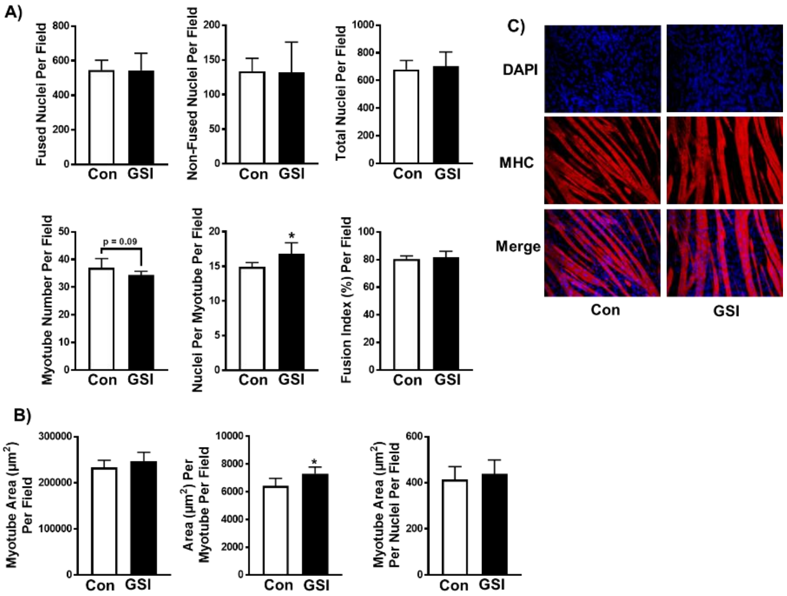

Figure S1. GSI augments myotube hypertrophy in day 6 myotubes. (**A**) Indices of myotube fusion. Graph order: Graph order: (top) Fused nuclei per field, Non-fused nuclei per field, Total nuclei per field, (bottom) Myotube number per field, Nuclei per myotube per field, Fusion index per field. (**B**) Indices of myotube hypertrophy. Graph order: Myotube area (μm) per field, Area (μm) per myotube per field, Myotube area (μm) per nuclei per field. (**C**) Representative image of 144-h myotubes co-stained with myosin heavy chain (MHC) and DAPI. Images were taken at 20x magnification and the scale bar = 50 μm. 120-h post differentiation C2C12 cells were treated every 12 h with either control (Con) or 4 μm γ-secretase inhibitor (GSI). Data were analyzed using a Student's T-test. * $p < 0.05$ vs. Con. ($n = 2$ experiments). Data are mean ± SD.

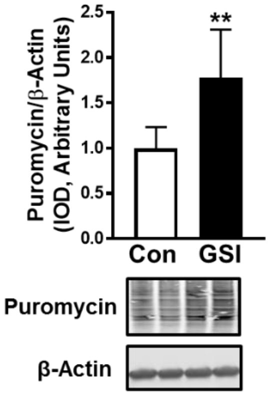

Figure S2. GSI augments protein synthesis in day 6 myotubes. Puromycin/β-Actin expression (Integrated optical density, IOD) in 144-h myotubes. 120-h post differentiation C2C12 cells were treated every 12 h with either control (Con) or 4 μm γ-secretase inhibitor (GSI). Thirty minutes prior to collection all cells were treated with 1 μm puromycin. For representative image: lanes 1 and 2 are

Con; lanes 3 and 4 are GSI. Data were analyzed using a Student's T-test. ** $p \leq 0.01$ vs. Control (Con) ($n = 3$ experiments). Data are mean ± SD.

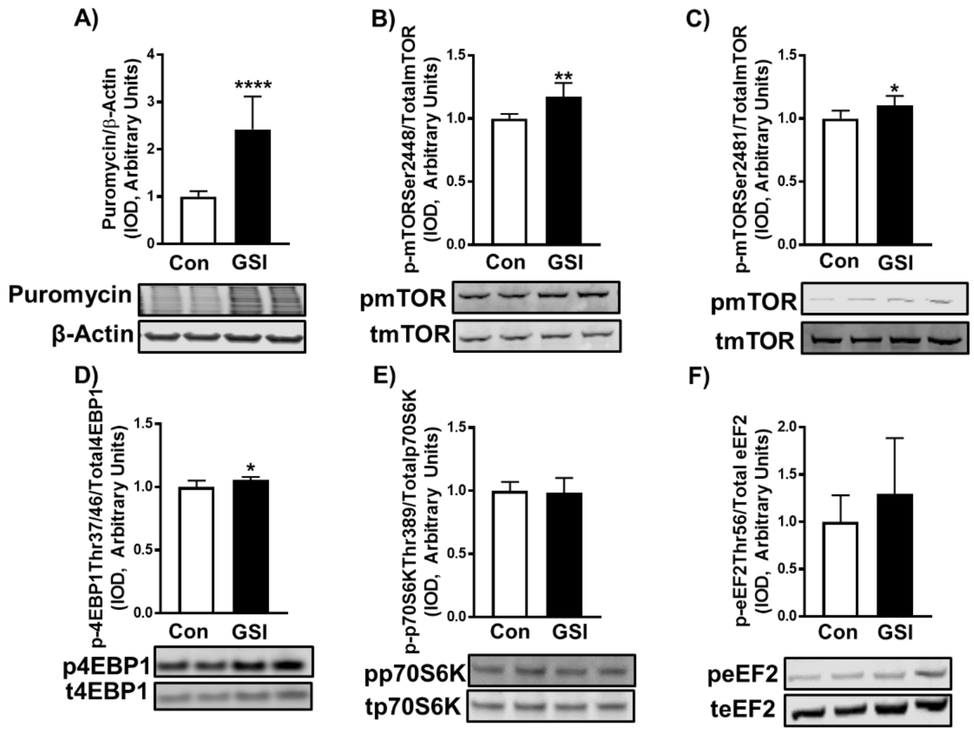

Figure S3. GSI elevates protein synthesis and mTOR signaling in C2C12 myoblasts. (**A**) Puromycin/β-Actin; (**B**) p-mTOR Ser2448/Total mTOR; (**C**) p-mTOR Ser2481/Total mTOR; (**D**) p-4EBP1 Thr37/46/Total 4EBP1; (**E**) pp70S6K Thr389/Total p70S6K; (**F**) p-eEF2 Thr56/Total eEF2 expression (Integrated optical density, IOD) in 48-h myoblasts treated with or without 4 µm γ-secretase inhibitor (GSI) every 12 h. Thirty minutes prior to collection all cells were treated with 1 µm puromycin. For representative image: lanes 1 and 2 are Con; lanes 3 and 4 are GSI. Data were analyzed using a Student's T-test. * $p \leq 0.05$ vs. Control (Con); ** $p < 0.01$ vs. Con; **** $p < 0.0001$ vs. Con ($n = 3$ experiments). Data are mean ± SD.

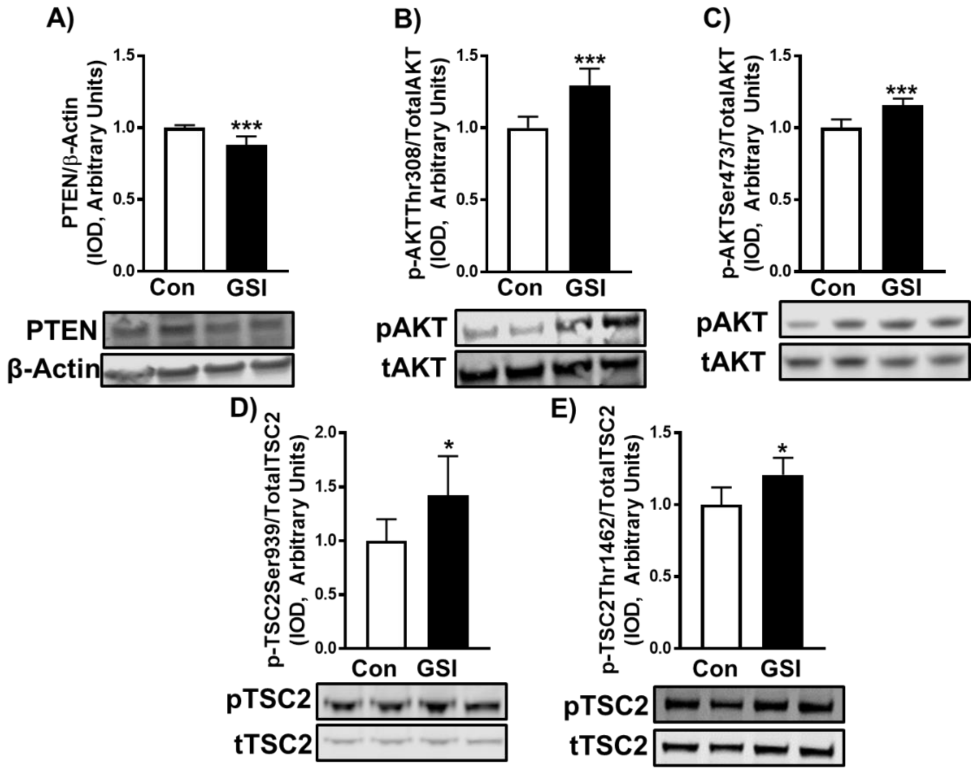

Figure S4. GSI modulates signaling upstream of mTOR in C2C12 myoblasts. (**A**) PTEN)/β-Actin; (**B**) p-AKT Thr308/Total AKT; (**C**) p-AKT Ser473/Total AKT; (**D**) p-TSC2 Ser939/Total TSC2; (**E**) p-TSC2 Thr1462/Total TSC2 expression (Integrated optical density, IOD) in 48-h myoblasts treated with or without 4 μm γ-secretase inhibitor (GSI) every 12 h. Thirty minutes prior to collection all cells were treated with 1 μm puromycin. For representative image: lanes 1 and 2 are Con; lanes 3 and 4 are GSI. Data were analyzed using a Student's T-test. * $p \leq 0.05$ vs. Control (Con); *** $p < 0.001$ vs. Con ($n = 3$ experiments). Data are mean ± SD.

© 2020 by the authors. Submitted for possible open access publication under the terms and conditions of the Creative Commons Attribution (CC BY) license (http://creativecommons.org/licenses/by/4.0/).
